# Unusual Spinal Foraminal Hemangioblastoma With Prominent Arteriovenous Shunt

**DOI:** 10.7759/cureus.46205

**Published:** 2023-09-29

**Authors:** Mariana Santos, Victor H Marussi, Christiane M Campos, Hugo Leonardo Doria-Netto, Ricardo Henrique Doria-Netto, Feres Chaddad-Neto, Lázaro Luís F Amaral

**Affiliations:** 1 Neuroradiology Department, Hospital de Braga, Braga, PRT; 2 Neuroradiology, Hospital da Beneficência Portuguesa de São Paulo, São Paulo, BRA; 3 Neurosurgery, Universidade Federal de São Paulo (UNIFESP), São Paulo, BRA; 4 Neurosurgery, Hospital da Beneficência Portuguesa de São Paulo, São Paulo, BRA; 5 Neurological Surgery, Universidade Federal De Sao Paulo (UNIFESP), Sao Paulo, BRA

**Keywords:** asl., schwannoma, spinal arteriovenous fistula, von hippel-lindau, hemangioblastoma

## Abstract

Von Hippel-Lindau (VHL) disease is a rare neurocutaneous disorder characterized by multiple benign and malignant tumors involving different organs (renal, adrenal, pancreas, liver, urogenital system, central nervous system, and head and neck region) due to mutations in the *VHL* tumor suppressor gene. Here, we describe a patient with unknown VHL disease who has complained of hypoesthesia of the right lower limb for about six years. A lumbar MRI was performed and revealed an expansive foraminal lesion at the right L3-L4 level and multiple serpiginous intradural and extramedullary flow voids involving the dorsal aspect of the spinal cord. The patient underwent digital subtraction angiography to exclude a spinal dural arteriovenous fistula, which revealed imaging features suggestive of spinal hemangioblastoma. In the presence of a spinal hemangioblastoma, a brain MRI was performed for further evaluation to rule out the possible diagnosis of VHL disease, and a solitary hemangioblastoma on the right cerebellar hemisphere was found. The patient underwent lumbar spine surgery, confirming the suspicious diagnosis of hemangioblastomas related to VHL disease.

## Introduction

Von Hippel-Lindau (VHL) disease is a rare neurocutaneous disorder whose prevalence is estimated to be between 1 in 36 000 and 1 in 50 000 individuals. It is characterized by multiple benign and malignant tumors due to mutations in the VHL tumor suppressor gene that can involve many compartments, such as the abdominopelvic, urogenital, and central nervous systems [1,2].

Hemangioblastoma is one of the manifestations of the disease, and it occurs in the intracranial compartment in most cases, mainly in the posterior fossa (44%-72%) and in the spinal cord (13%-59%) [1]. Spinal hemangioblastoma accounts for 2.1% of all spinal tumors [3]. We present a case of spinal hemangioblastoma in the lumbar region with foraminal extension, mimicking a schwannoma and a spinal arteriovenous fistula due to prominent and longitudinally extended flow voids in the dorsal aspect of the spinal cord.

## Case presentation

**Figure 1:**
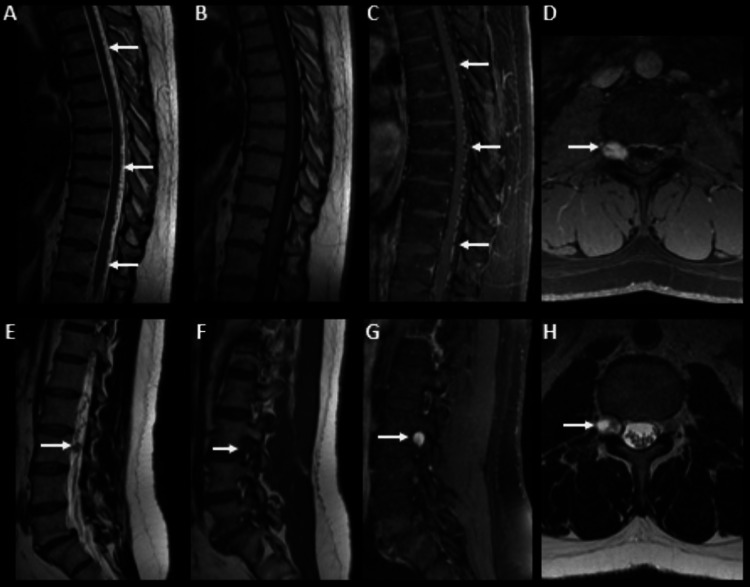
Magnetic resonance imaging of the lumbar spine (sagittal and axial views). Sagittal T2WI (A) of the thoracic spine shows multiple serpiginous flow voids (arrows), located in the dorsal aspect of the spinal cord, difficult to identify on sagittal T1WI (B), revealing intensely gadolinium-enhancement on sagittal T1WI fat-saturated (C, arrows). Sagittal T2WI of the lumbar spine (E) reveals caudal extension of the lesions, with a nodular lesion at L3 level (arrow) that extended to the right foraminal foramen, showing hypointense signal on sagittal T1WI (F), intense homogeneous enhancement after gadolinium (on axial (D) and sagittal (G) T1WI fat-saturated), and heterogeneous signal on axial T2WI (H, arrow).

A 47-year-old man with no relevant personal medical history presented with hypoesthesia on the lateral side of the right lower limb for about six years, worsening in recent months, without motor deficits or sphincter alterations. The physical examination was unremarkable. Thoracic spine magnetic resonance imaging (MRI) (Figure 1) revealed multiple prominent serpiginous intradural and extramedullary flow voids on T2-weighted images, involving predominantly the dorsal aspect of the spinal cord, from the upper thoracic segment to L3, with no intramedullary signal changes, suggesting edema. On post-contrast T1-weighted images, there were serpentine-enhancing vessels on the cord surface, with a round-enhancing foraminal lesion at the right L3-L4 level.

**Figure 2:**
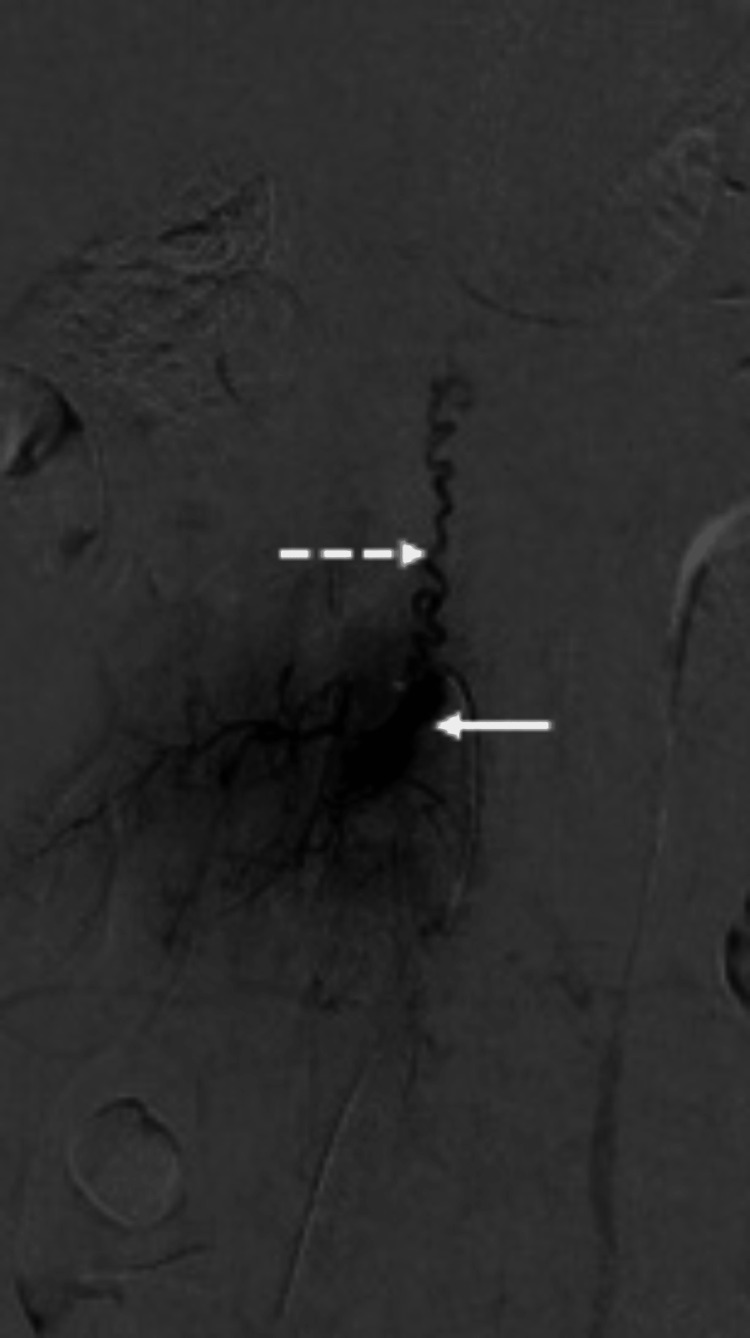
Digital subtraction angiography of the lumbar spine. Digital subtraction angiography (DSA) right L3 selective lumbar artery contrast injection confirmed hypervascularized expansive lesion at the right L3-L4 level, with hypertrophy of radiculomeningeal arteries located in the intracanal compartment with foraminal extension (arrow). Note the enlargement of the corresponding radicular vein, exhibiting an ascending course connecting the medullary veins (anterior and posterior) (dashed arrow).

The patient underwent digital subtraction angiography (Figure 2), which revealed a densely enhancing lesion with associated dilated arteries and prominent draining veins, features characteristic of a hemangioblastoma.

**Figure 3:**
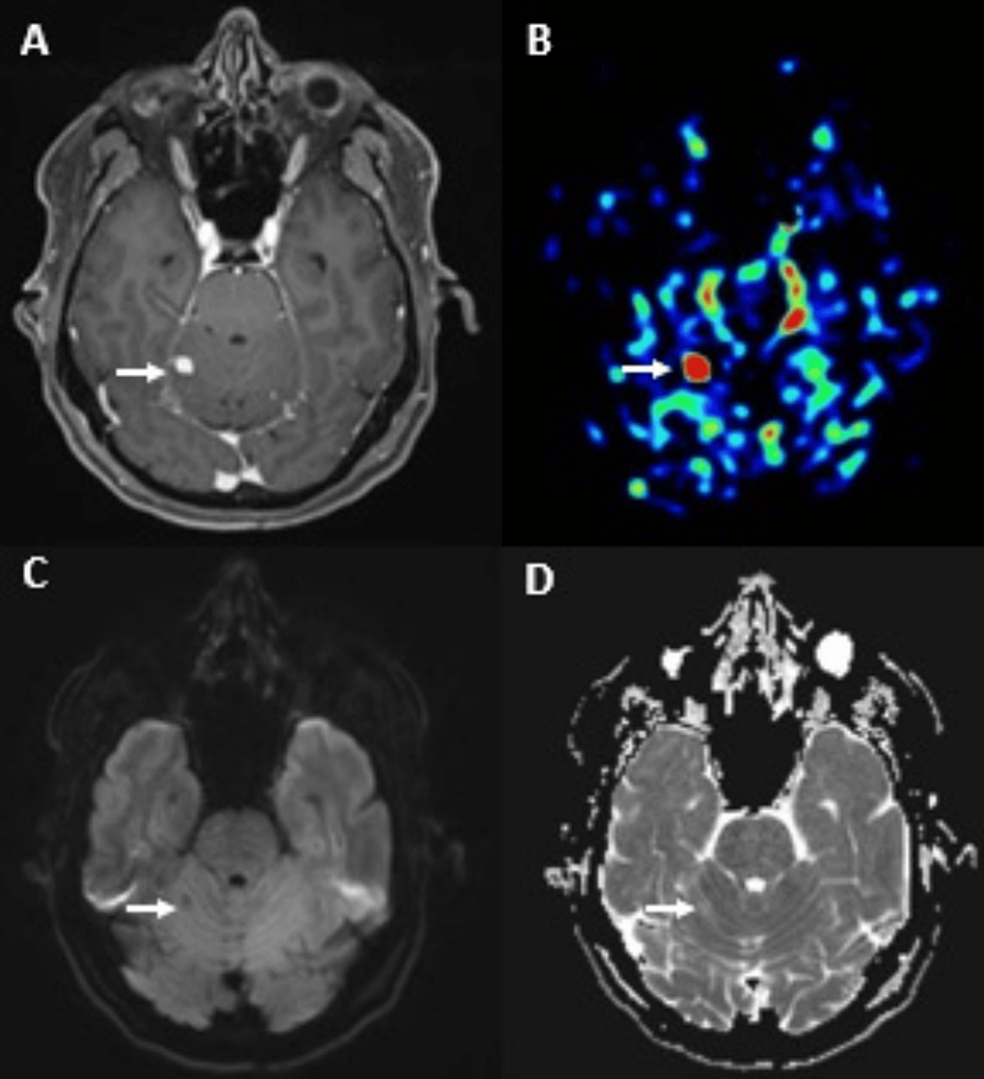
Brain magnetic resonance imaging (axial view). (A) Axial T1WI of brain MRI shows an intra-axial nodular lesion with homogeneous enhancement in the right cerebellar hemisphere (arrow) and increased cerebral blood flow (CBF) (B). Axial DWI shows a low signal (C) and a corresponding apparent diffusion coefficient (ADC) map (D) showing increased ADC of the nodule (arrow), which is very suggestive of cerebellar hemangioblastoma.

Due to the suspicion that it could be a hemangioblastoma. A brain MRI (Figure 3) was performed for further evaluation and depicted a solitary small nodular cortico-pial-enhancing lesion on the right cerebellar hemisphere without restricted diffusion and high tumor blood flow in arterial spin labeling (ASL) perfusion. These findings suggested hemangioblastomas, and the patient was screened for Von Hippel-Lindau syndrome, which was confirmed.

**Figure 4:**
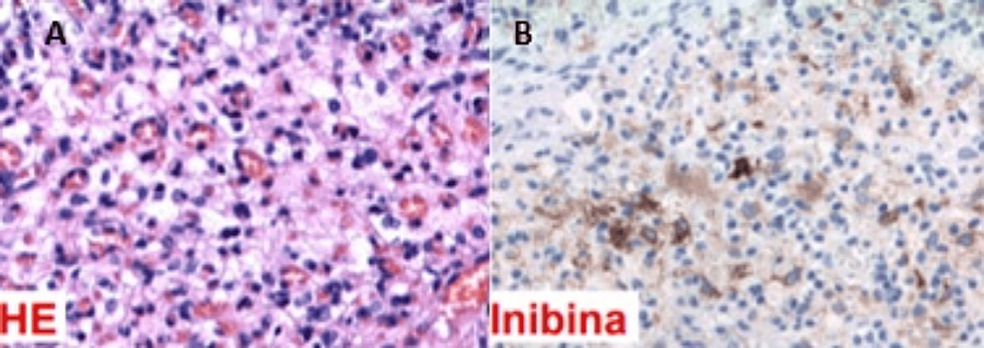
Histological findings from a foraminal spine lesion. Histology examination shows compact stromal cells with numerous associated capillaries (A) (HE, Gx25), and an immunohistochemistry study (B) depicts positivity for alfa-inhibin and S-100 protein.

The patient underwent lumbar spine surgery through a posterior approach with laminotomy and foraminotomy at right L3-L4 level, confirming an hypervascularized expansive lesion with extradural and intradural components, causing compression of the roots of the cauda equina. Under optical microscopy, complete resection of the lesion was performed, with dural resection. The homeostasis review was carried out with two units of Superclot®, an absorbable hemostatic system to control bleeding. Hermetic dural synthesis under the microscope was performed, as well as replacement and bone fixation of the laminotomy with plates and screws. Intraoperative neurophysiological monitoring with an electrode to root stimulation was performed to ensure the preservation of root function during tumor excision. Histology examination revealed compact stromal cells with numerous associated capillaries (Figure 4a), and an immunohistochemistry study depicted positivity for alfa-inhibin and S-100 protein (Figure 4b), which corroborates the suspicious diagnosis of hemangioblastomas related to VHL disease.

**Figure 5:**
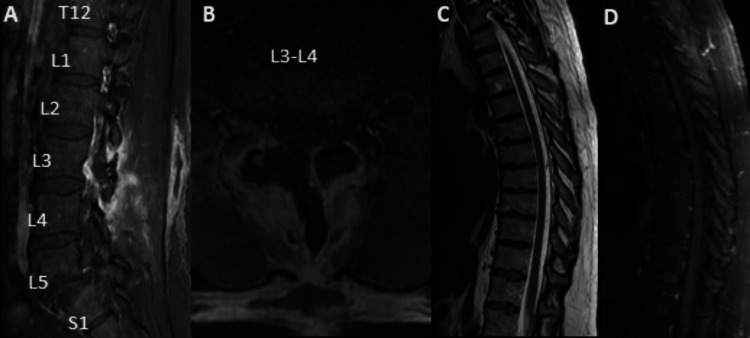
Post-operative magnetic resonance imaging of the lumbar spine (sagittal and axial views). Sagittal (A) and axial (B) T1WI of the lumbar spine show post-operative imaging findings, with complete resection of the right foraminal hemangioblastoma at L3-L4 level. Sagittal T2WI (C) and sagittal T1WI fat-saturated (D) of the thoracic spine depict the absence of flow voids in the dorsal aspect of the spinal cord.

**Figure 6:**
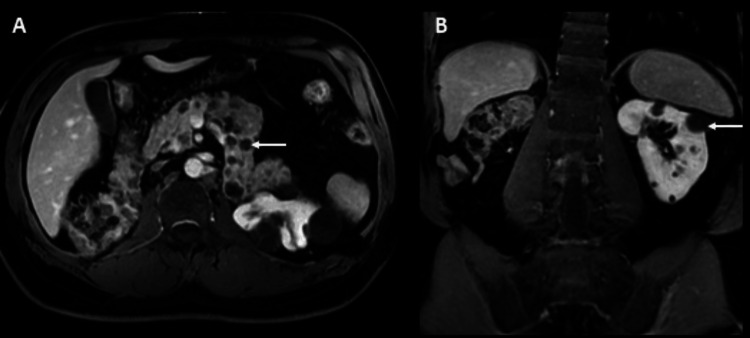
Abdominal magnetic resonance imaging (axial and coronal views). Axial (A) and sagittal T1WI fat-saturated with gadolinium show multiple pancreatic (A) and renal (B) cysts (arrows).

Fourteen months later, the patient performed a follow-up lumbar spine MRI (Figure 5), demonstrating post-surgical manifestations with total resection of the hemangioblastoma at the L3-L4 level and absence of flow voids on the dorsal aspect of the spinal cord. Multiple pancreatic and renal cysts were seen, relating to the underlying disease. The patient has made significant improvements in his neurological state. Figure 6 shows abdominal MRI.

## Discussion

Spinal hemangioblastomas are rare, representing 2.1% of all spinal tumors, and are mostly sporadic and usually present in the fourth decade [1]. One-third of patients with spinal or cerebral hemangioblastomas have Von Hippel-Lindau disease, and in these patients, other manifestations of the central nervous system must be looked at, namely retinal hemangioblastomas, paragangliomas, and endolymphatic sac tumors [1,2].

The most common location for hemangioblastomas is the thoracic cord, and the majority of these lesions have an intramedullary component, exhibiting eccentric growth and, sometimes, an exophytic component (most commonly, along the dorsum of the cord) [4,5].

Although the literature reports entirely extradural or extra- and intradural hemangioblastomas, they are considered to be a very rare feature, making this diagnosis particularly challenging [2,6]. To our knowledge, there are few cases described in the literature reporting extradural foraminal hemangioblastoma in a patient with Von Hippel-Lindau disease, showing prominent vessels and mimicking spinal dural arteriovenous fistula and schwannoma [7]. Aytar et al. [8] and Mariniello et al. [9] reviewed the literature, and the majority of extradural hemangioblastomas were located in the thoracic spine (40%), followed by the lumbar (33%) and cervical spine (13%). The differential diagnosis of a foraminal lesion should include a neurogenic tumor (schwannoma or neurofibroma). However, the presence of signs of a hypervascularized lesion with an arteriovenous shunt should be a "red flag" to consider the hypothesis of hemangioblastoma. The diagnosis of spinal dural arteriovenous fistula was excluded by the presence of a vascularized mass and the absence of edema in the conus medullaris.

In these challenging cases, a brain MRI must be performed to exclude other potential differential diagnoses. Our case demonstrates the role of the brain MRI in establishing the diagnosis of hemangioblastoma in the context of VHL disease. The presence of flow voids in and around the tumor, the low signal on the diffusion-weighted image (DWI), the strong homogenous enhancement after gadolinium, and the high rate of tumor blood flow in the ASL technique suggested this diagnosis [10]. Owing to the risk of perioperative bleeding, presurgical diagnosis is particularly important as it allows clinicians to select the optimal management approach for these particular lesions.

Microsurgical total resection is the treatment of choice for symptomatic spinal hemangioblastomas because a good prognosis may be expected after successful surgery with clinical improvement [11]. The recurrence of these lesions is mainly associated with subtotal resection, being between 6.25 and 20% in sporadic hemangioblastomas and more frequent in patients with VHL disease [6,12]. Therefore, close follow-up MRIs are needed, mainly in patients with VHL disease, where recurrence can occur even after complete resection of the lesion for a long period without recurrence [12].

## Conclusions

The present case represents atypical imaging findings of spinal hemangioblastoma, a hypervascularized tumor that can cause an arteriovenous shunt, mimicking a spinal dural arteriovenous fistula with schwannoma due to the foraminal extension, in a patient with Von Hippel-Lindau disease. In these challenging cases, because many other entities can present similar radiological imaging, investigation with other complementary means of diagnosis should be performed in order to establish the correct diagnosis and define the best therapeutic approach for the patient.
